# Ultrasound Elastography Assessment of Knee Intra-Articular Adhesions at Varying Knee Angles

**DOI:** 10.3390/bioengineering11070706

**Published:** 2024-07-12

**Authors:** Jiling Ye, Linjing Peng, Angang Ding, Shijie Chen, Bin Cai, Yifei Yao

**Affiliations:** 1 Rehabilitation Department, Shanghai Ninth People’s Hospital, Shanghai Jiao Tong University School of Medicine, Shanghai 200023, China; jiling_ye@163.com; 2 Med-X Research Institute, School of Biomedical Engineering, Shanghai Jiao Tong University, Shanghai 200030, China; penglinjing1995@sjtu.edu.cn; 3 Ultrasound Medicine Department, Shanghai Ninth People’s Hospital, Shanghai Jiao Tong University School of Medicine, Shanghai 200023, China; dingangang1205@163.com; 4 Department of Computer Science, School of Computing, Tokyo Institute of Technology, Ookayama, Meguro-ku, Tokyo 152-8550, Japan; chen@cb.cs.titech.ac.jp

**Keywords:** knee adhesion, scar, ultrasound elastography, shear wave elastography

## Abstract

We aimed to verify the feasibility of using shear wave elastography (SWE) to quantify knee scars and the elastic modulus of scar tissues. Overall, 16 participants underwent SWE assessments and range-of-motion measurement and completed the Knee Injury and Osteoarthritis Outcome Score. The inter-rater reliability for SWE in the suprapatellar bursa, below the patellar tendon, and in the medial and lateral trochlear groove remained within 0.861–0.907. The SWE values in the four regions increased with increasing knee angle, and significant differences were observed between the values for below the patellar tendon and the suprapatellar bursa at knee flexion angles of 60° and 90°. The SWE values of the medial and lateral trochlear groove at 30°, 60°, and 90° knee flexion were higher on the affected side. A negative correlation was observed between the SWE values for the lateral trochlear groove at 0°, 30°, and 60° and those for below the patellar tendon at 0° and the suprapatellar bursa at 30° with both active and passive knee extension. The suprapatellar bursa value at 60° exhibited a positive correlation with both knee flexion and passive knee flexion, whereas that of the suprapatellar bursa at 90° exhibited a positive correlation with both the range of motion and passive range of motion. SWE is a replicable and effective method for detecting scar strength in the knee joint.

## 1. Introduction

Intra-articular adhesions are common complications of knee disease and injury, and the probability of developing adhesions after knee surgery is 2.0–6.0% [1,2,3,4]. The main clinical manifestations, such as limited knee flexion and extension, decreased accessory joint motion, and chronic knee pain [5], affect the quality of life of patients [6], making them less satisfied with the consulting physicians and treatment.

Previous studies attribute this pathological condition to inter-tissue scar proliferation and contracture [7,8]. Scar tissue is fibrous tissue usually formed during wound healing. Postoperative trauma in knee joint structures, including the suprapatellar capsule, between the patella and the femoral condyle, and below the proximal quadriceps and the patellar tendon, causes fibrosis and scar tissue formation. This extensive fibrotic reaction leads to almost complete filling of the tissue structures of the knee joint with dense scar tissue, thereby limiting the physiological and accessory movements of the joint and leading to joint adhesions [7,9,10]. Currently, patients with severe joint adhesions are mainly treated with manipulation under anaesthesia or arthroscopic lysis [6,11]. Magnetic resonance imaging (MRI) and ultrasound findings in patients with knee adhesions show that scar tissue fills the internal joint space (mainly the suprapatellar bursa [SPB], the trochlear groove, and below the patellar tendon [BPT]); this can restrict the mutual sliding and rotation of bony structures and lead to compromised movement [12]. Owing to the strong correlation between scar tissue and knee adhesions, it is important to determine the in vivo mechanical properties of scar tissues in patients with adhesions and observe the changes in tension during knee joint motion to establish an appropriate treatment strategy and evaluate clinical treatment outcomes. MRI can help detect and measure the thickness of intra-articular adhesions, but MRI cannot help measure their intensity or measure them from multiple angles [13].

Ultrasound elastography is an emerging technique that was first proposed in 1991 [14]. Compression elastography is based on the principle of the strain generated by the compression of superficial tissue. It calculates the real-time displacement difference between different tissues by repeatedly compressing them using an ultrasound transducer; this helps determine the difference in elasticity between the lesion and the surrounding normal tissue. However, this technique has drawbacks, including the need to maintain the probe perpendicular to the tissue and manually adjust the compression force used to measure the tissue strain. Compared with conventional ultrasound elastography, shear wave elastography (SWE) has the advantage of measuring the elastic modulus of tissues quickly, conveniently, and in real time [15]. SWE can accurately assess the elastic characteristics of in vivo tissues and their local lesions, has broad prospective applications in clinical practice, and has been widely used in many fields [16,17,18]. However, this technique has not yet been applied to investigating the in vivo mechanical properties of knee adhesions.

To the best of our knowledge, this study is the first to use SWE to measure joint adhesions after knee trauma. We aimed to verify the feasibility of using SWE to quantify knee joint scars and the elastic modulus of knee scar tissues in patients with joint adhesions after knee trauma, thereby laying the foundation for the precise evaluation and treatment of knee adhesions.

## 2. Materials and Methods

### 2.1. Study Design

This cross-sectional, exploratory study was approved by the Shanghai Ninth People’s Hospital, Shanghai Jiao Tong University School of Medicine Ethics Committee (SH9H-2021-T365-1) and was conducted in accordance with the Declaration of Helsinki.

### 2.2. Participants

Participants were recruited between December 2021 and November 2022 from the rehabilitation department of our hospital. Written informed consent was obtained from each participant. The inclusion criteria were as follows: age, >16 years; duration, 0–6 months after knee trauma or surgery; and range of motion (ROM), <110°. The exclusion criteria were skin damage, limited movement due to nerve injury, and joint infection.

### 2.3. Subjective Assessments

#### 2.3.1. SWE

Sonographic examinations were performed by a single radiologist with >10 years of experience in musculoskeletal sonography. All examinations were performed using the Aixplorer V scanner (SuperSonic Imagine, Aix-en-Provence, France).

B-mode and SWE assessments were performed using a 15-4 MHz linear transducer that produces acoustic radiation force impulse pulses. SWE measurements of the knee adhesions were obtained in the SPB, in the medial (MTG) and lateral trochlear groove (LTG), and BPT at 0°, 30°, 60°, and 90° knee flexion, respectively.

**Figure 1:**
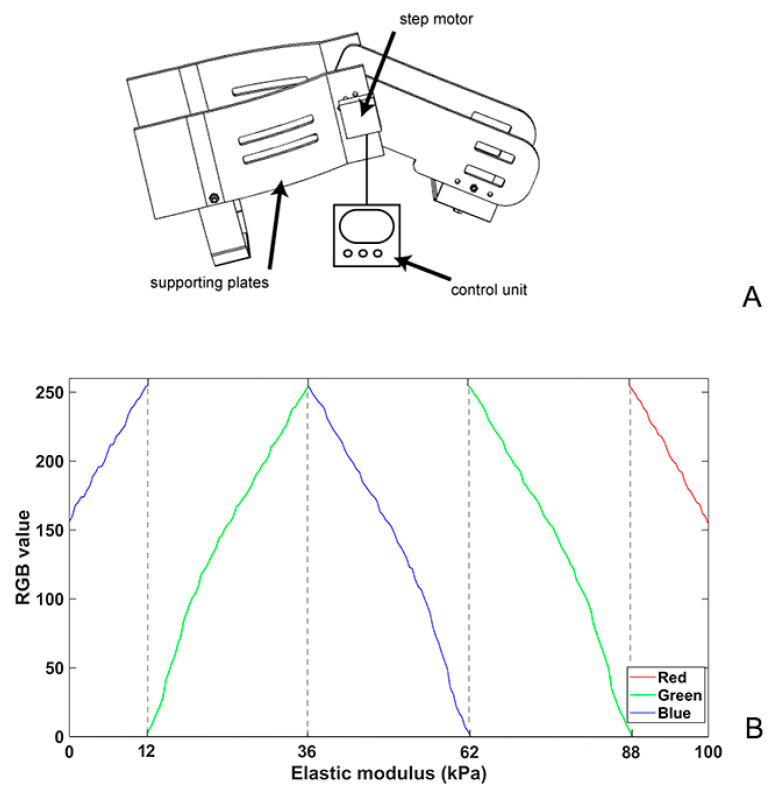
Schematic of a customised device for the different patients’ static knee flexion angles (**A**) and the relationship between RGB values and elastic modulus per pixel for calculating ROI in SWE (**B**). SWE, shear wave elastography.

**Figure 2:**
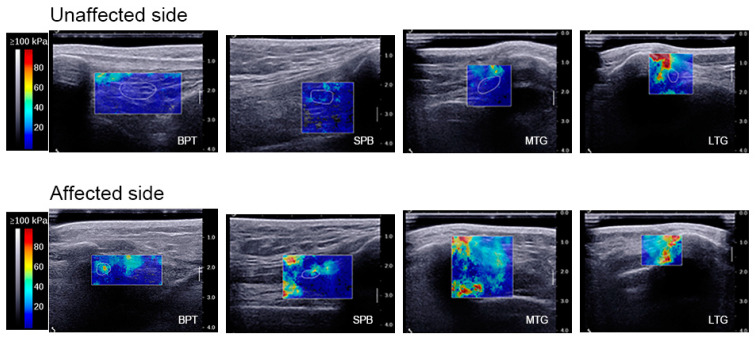
Elastographic imaging. Elastographic imaging at 0° knee flexion: analysis of BPT, the SPB, the MTG, and the LTG on the unaffected side and affected side. The scar tissue was delineated by a white line (i.e., ROI) on the affected side, whereas the corresponding anatomical region was outlined on the unaffected side. Within the ROI, the colour map showed the relative SWE at each location in order of increasing value: blue, green, yellow, and red. BPT, below the patellar tendon; SPB, suprapatellar bursa; MTG, medial trochlear groove; LTG, lateral trochlear groove; ROI, region of interest.

A self-designed device which could maintain static knee flexion at different angles was developed (Figure 1A). The apparatus comprised a step motor (42BYGH34-401A; Yuhui, China) controlled by a motor driver (TB6600; Yuhui, China), an alternating current signal generator, and four 3D-printed acrylonitrile butadiene styrene supporting plates. The thigh and calf plates were connected using gears, and the bilateral supporting plates were connected using elastic straps. Minimal contact pressure was applied during the data acquisition to minimise the axial pre-load, which was confirmed by applying the minimal amount of pressure required to generate an image and avoid visible distortion of the superficial skin and fascia. The SWE values were displayed as either a colourised map or split greyscale image depicting the anatomy, and a split-screen display was used to eliminate anatomical ambiguity when placing the sample locations. In the colour-mapped SWE image, red and blue pixels represent regions of stiffer and softer tissue, respectively. The SWE value corresponding to each pixel within the region of interest (ROI) was primarily influenced by one of the RGB parameters in the five regions, as shown in the Figure 1B. The piecewise function described the relationship between the SWE value and the RGB values, enabling the estimation of an irregular ROI, specifically identifying scar tissue located in different anatomical regions at varying knee angles. The average SWE values within the ROI, ranging from 0 to 100 kPa, were obtained by manually outlining the boundary and were derived from raw radiofrequency data using a customised programme, as shown in Figure 2.

#### 2.3.2. Knee ROM

Knee joint ROM was measured using static photographs of the knee joint. During measurement, the participants flexed and extended their knees in the supine position, and the lateral epicondyle of the femur was considered the axis, with the greater trochanter of the femur and the lateral malleolus representing the fixed and moving points, respectively. These points were pre-marked on the body surface [19]. ROM includes active and passive motion; knee extension was measured by maximally extending the knee joint and was defined as 0° extension [20]. Knee flexion was measured by bending the knee and gliding the heel as far as possible toward the buttocks [20].

#### 2.3.3. Knee Injury and Osteoarthritis Outcome Score (KOOS)

The KOOS is a patient-reported outcome measure intended for young, middle-aged, and older adults with knee injury and/or knee osteoarthritis, and it can be used to monitor the disease course and outcomes following surgical, pharmacological, and other interventions [21]. The KOOS has five subscales: pain (9 items), other symptoms (7 items), activities of daily living (17 items), sports and recreation function (5 items), and knee-related quality of life (4 items). Each subscale is separately scored from 0 (extreme knee problems) to 100 (no knee problems). The KOOS has confidence regarding its content validity, internal consistency, test–retest reliability, construct validity, and responsiveness to age- and condition-relevant subscales [22]. Participants completed the KOOS on the day of the SWE evaluation.

#### 2.3.4. The ICC Test

During SWE assessment, the agreement among three independent observers was calculated for the four anatomical regions (BPT, the SPB, the MTG, and the LTG) in ultrasound images. Three observers participated in a unified training session for ultrasound imaging and ROI selection. A score of <0.40 was regarded as poor, 0.40–0.75 as fair to good, and >0.75 as excellent agreement among the three observers.

### 2.4. Data Analysis

The sample size was calculated with an α-value of 0.05 for a 95% confidence level; we aimed for a power of 80% (1-β). Data management and statistical analyses were performed using IBM SPSS (version 22.0; IBM Corp., Armonk, NY, USA) and R (4.2.2). Quantitative data with a normal distribution are presented as means ± standard deviation ($\bar{x}$ ± s), whereas non-normally distributed data are described as means (Q1, Q3). Qualitative data are presented as percentages. Given the presence of repeated measures in our study, we employed a mixed-effects model to analyse the data and perform pairwise comparisons. The correlation analysis between SWE, ROM, and the KOOS was conducted using Pearson’s correlation, and R and ggplot2 (3.3.6) were used to clean and visualise the data. A two-way random model was selected to calculate the ICC of the SWE values; the results of single measures represented the ICC. All the statistical tests were two-sided, with a *p*-value of <0.05 indicating statistical significance.

## 3. Results

A total of 32 lower limbs from 16 patients with knee adhesions were included in the survey, consisting of 16 unaffected lower limbs and 16 affected lower limbs. The patients’ demographic characteristics are summarised in Table 1; there was no significant difference in the demographic characteristics of the patients. The inter-rater reliability for measuring the SWE values shown in Table 2 was within 0.861–0.907 for BPT, the SPB, the MTG, and the LTG, indicating excellent reliability.

**Figure 3:**
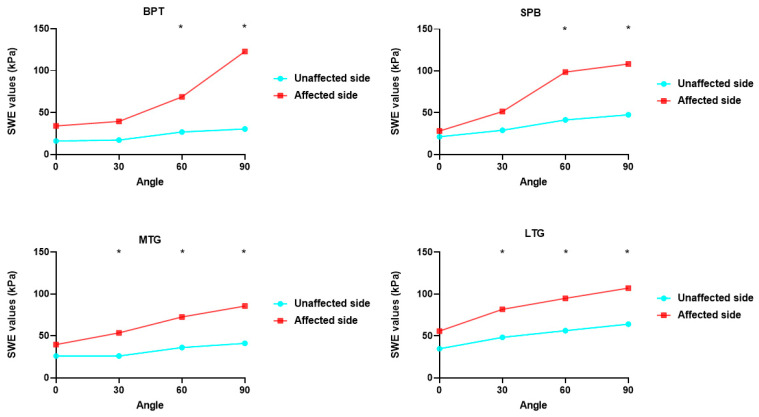
Comparison of SWE values. Comparison of SWE values of the four parts of the knee at different angles. SWE, shear wave elastography. * *p* < 0.05.

**Figure 4:**
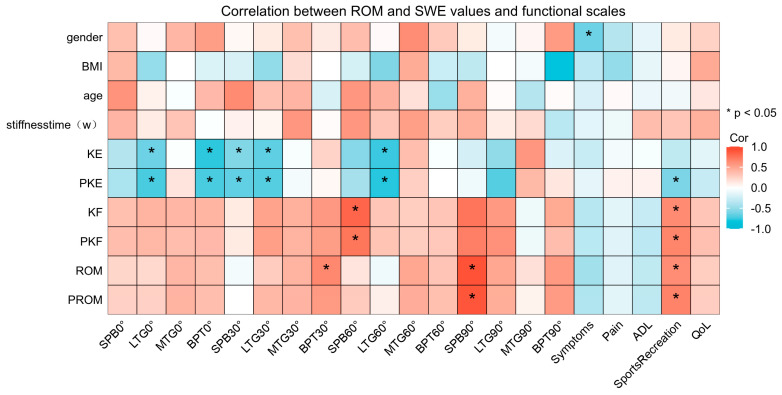
Correlation between range of motion and SWE values and functional scales. SWE, shear wave elastography.

The ROM and SWE values from various angles and functional scales are shown in Table 3, and the SWE values of the four knee parts at different angles are compared in Figure 3. The SWE values in the four regions increased with increasing knee angle, and significant differences were observed between the SWE values of BPT and the SPB at knee flexion angles of 60° and 90°. The SWE values of the MTG and the LTG at 30°, 60°, and 90° knee flexion were significantly higher on the affected side than on the unaffected side. The correlation between ROM, the affected-side SWE values, and the functional scale scores is shown in Figure 4. The correlation heat map revealed a significant negative correlation between the SWE values for the LTG at 0°, 30°, and 60° and BPT at 0° and the SPB at 30° for both active and passive knee extension. Moreover, the SWE values for the SPB at 60° exhibited a significant positive correlation with both active and passive knee flexion, whereas the SWE values for the SPB at 90° exhibited a significant positive correlation with both ROM and passive ROM.

## 4. Discussion

Knee adhesions are common after surgery or trauma and often lead to loss of knee function or chronic joint pain. They may even cause degenerative articular cartilage disease and joint stiffness [23], thereby imposing a significant physical and economic burden on patients. Articular adhesions are classified as extra- or intra-articular [24]. Intra-articular lesions include plasma fibrous exudates, blood accumulation in the joint cavity, hematoma mechanisation, and adhesion formation; extra-articular lesions include adhesions of the quadriceps muscle, adhesions between the middle femoral muscle and the periosteum of the femur, and adhesions of the SPB [25]. Nicoll et al. [7] stated that scar tissue and tissue contracture proliferation are essential causes of adhesions in joints. Large amounts of hyperplastic scar tissue are widely distributed among the various tissue structures, limiting the mutual sliding of the structures and causing contractures of the joint capsule and extra-articular tendons [9,11]. Considering the close association between scar growth and joint adhesions, continuously improving our knowledge and understanding of scar tissue is valuable as a guide to preventing and treating joint adhesions. The biomechanical characteristics of scar tissue in knee adhesions have not been explored; therefore, determining the mechanical properties of scar tissues is of great theoretical and practical importance in both biomechanical and clinical research.

Herein, the biomechanical characteristics of the scar tissues in patients with knee adhesions were measured in four positions, including knee extension and 30°, 60°, and 90° flexed knee positions, within four regions, including the suprapatellar capsule, BPT, the MTG, and the LTG. The intensity of the scar elastic modulus, the knee scar geometry, and the average scar strain during knee flexion and extension were determined, and the association between the scar elastic modulus, ROM, and the KOOS was analysed. The results of our study reflect those of a cross-sectional study [26] that measured the elastic modulus of pathological scars; a correlation between the elastic modulus of pathological scars and the severity of clinical symptoms was confirmed. However, a study by Kawai et al. [27] investigating the relationship between the length and area of the muscle scar tissue and fascial stiffness in patients with hamstring strains found no significant association between the two parameters. A literature review demonstrated relatively few relevant studies, which focused primarily on conditions including burn injuries [28], muscle strains [29], and Achilles tendon repair [30]. Future studies are required to validate the association between the biomechanical properties of scar tissue and the functional performance of knee adhesions.

This study clarified the scar strength in patients with knee adhesions. We found that the elastic modulus was significantly higher on the affected side than on the unaffected side, suggesting that scar tissue plays a crucial role in the process of joint adhesions and that biomechanical factors positively influence scar proliferation. However, the mechanism underlying scar tissue induction has not been fully elucidated. Although there are many theories [31], none are generally accepted either nationally or internationally. Mechanical theory, which has recently gained attention [32], states that the tensional force at the wound edges is one of the main factors stimulating scar proliferation. Under tension at the wound site, the metabolic function of collagen is disturbed, and it continues to multiply abnormally and rapidly; this leads to uncontrolled overgrowth and subsequent scar formation [33,34]. Thus, biomechanical factors likely promote scar proliferation. Small leucine-rich proteoglycans link and interact with collagen fibrils [29]; this interaction plays a role in regulating the tendon structure and biomechanics, improving fibrovascular scar tissue proliferation and promoting tendon healing. Further longitudinal studies that investigate the changes in scar tissue during scar proliferation and provide a preliminary theoretical basis for the prevention and treatment of knee adhesions are warranted.

Commonly used methods to determine the mechanical properties of biological tissues include MRI [35] and ultrasonography [36]. In this study, SWE was used for the first time to measure the elastic modulus of scars in patients with post-traumatic joint adhesions of the knee in varying angles. SWE is currently the newest ultrasound elastography technique and works by applying a pulsed force to the tissue using a fixed probe, subsequently generating shear waves in tissues. Shear waves are transverse waves generated in an elastic medium subjected to periodic shear that propagates through the adjacent tissues in a transverse plane perpendicular to the primary wave [37]. Based on the shear wave velocity, the elastic modulus value was calculated according to Young’s modulus formula, thus providing a quantitative description of the mechanical properties of the scar tissues. The use of a fixed probe eliminates the need for manual pressure application, eliminates operator errors, reduces dependence on the examiner, and improves the repeatability of operation. We found the elastic modulus of the scar tissue on the affected side to be significantly higher than that on the unaffected side; therefore, SWE can reflect the severity of scar adhesions and may be a reliable tool for assessing the treatment outcomes of patients with joint adhesions.

This study has some limitations. Although the sample size of this study is sufficient, it is still a small sample. This study included a variety of surgical types, although all were intra-articular surgeries. And this article mainly discusses the intensity of the scars but does not discuss the volume of the scars. We will further elucidate these factors through regression analysis in subsequent research. This was a cross-sectional study; thus, the changes in the biomechanical characteristics of scar tissue during the formation of joint adhesions require further investigation. In the future, we hope to investigate the change in scar intensity before and after rehabilitation, as well as determining the relationship between rehabilitation success, failure, and scar intensity.

## 5. Conclusions

This is the first study to quantitatively characterise the biomechanical properties of scar tissue in patients with knee adhesions. The technical advantages of SWE were fully utilised for the mechanical measurement of scar tissue in patients with knee adhesions, and pre-set brace stops were used to fix the patients’ static angles during the ROM measurements to reduce testing errors. SWE provides the possibility of quantification for the assessment of patients with knee adhesions.

## Figures and Tables

**Table 1:** Demographic and descriptive characteristics of the participants (*n* = 16).

Parameters	
Male	7
Female	9
Age (years)	30.3 ± 10.7
Height (cm)	169.2 ± 8.7
BMI (kg/m^2^)	22.4 ± 3.1
Time of adhesion (weeks)	14.8 ± 5.7
ACLR	8
PCLR	2
Patellar dislocation	2
Meniscus repair	2
ACLR + MCLR	1
Patellar fracture	1

ACLR, anterior cruciate ligament reconstruction; PCLR, posterior cruciate ligament reconstruction; MCLR, medial collateral ligament reconstruction; BMI, body mass index. Data are presented as n or means ± standard deviation.

**Table 2:** ICC test for inter-rater reliability for measuring SWE values.

	Inter-Rater Reliability
BPT	0.907
SPB	0.861
MTG	0.899
LTG	0.897

SWE, shear wave elastography; BPT, below the patellar tendon; SPB, suprapatellar bursa; MTG, medial trochlear groove; LTG, lateral trochlear groove.

**Table 3:** ROM, SWE values, and functional scales (*n* = 16).

Parameters		Unaffected Side	Affected Side
KE		2.92 ± 2.94	−9.83 ± 7.94
PKE		5.33 ± 3.60	−5.42 ± 7.14
KF		135.67 ± 8.02	70.58 ± 24.02
PKF		142.08 ± 8.69	76.75 ± 26.04
ROM		138.58 ± 7.49	60.75 ± 23.81
PROM		147.42 ± 9.17	71.33 ± 25.74
Symptoms		67.86 ± 15.00	
Pain		28.24 ± 13.29	
ADL		27.45 ± 12.23	
Sports Reactions		17.50 ± 18.03	
QoL		19.79 ± 13.80	
SWE values of BPT (kPa)	0° KF	16.04 ± 11.84	34.04 ± 11.52
	30° KF	17.13 ± 13.75	39.41 ± 18.38
	60° KF	26.76 ± 21.96	68.63 ± 33.63
	90° KF	30.46 ± 23.51	122.93 ± 42.50
SWE values of the SPB (kPa)	0° KF	21.35 ± 12.96	28.21 ± 11.41
	30° KF	29.00 ± 10.81	51.49 ± 19.96
	60° KF	41.44 ± 29.32	98.74 ± 47.27
	90° KF	47.52 ± 16.18	108.32 ± 45.25
SWE values of the MTG (kPa)	0° KF	25.98 ± 13.90	39.57 ± 14.80
	30° KF	25.92 ± 7.30	53.62 ± 18.58
	60° KF	36.06 ± 13.88	72.60 ± 30.68
	90° KF	41.05 ± 13.65	85.58 ± 20.40
SWE values of the LTG (kPa)	0° KF	34.68 ± 15.24	55.82 ± 20.93
	30° KF	48.25 ± 26.16	81.77 ± 31.97
	60° KF	56.34 ± 25.12	94.92 ± 34.93
	90° KF	64.00 ± 31.86	107.19 ± 35.71

Data are presented as means ± standard deviation. KE, knee extension; PKE, passive knee extension; KF, knee flexion; PKF, passive knee flexion; PROM, passive range of motion; ROM, range of motion; ADL, activities of daily living; QoL, quality of life; SWE, shear wave elastography; BPT, below the patellar tendon; SPB, suprapatellar bursa; MTG, medial trochlear groove; LTG, lateral trochlear groove.

## Data Availability

The raw data supporting the conclusions of this article will be made available by the authors on request.
